# Roles of renin-angiotensin system and Wnt pathway in aging-related phenotypes

**DOI:** 10.1186/s41232-016-0018-1

**Published:** 2016-08-09

**Authors:** Takehiro Kamo, Hiroshi Akazawa, Jun-ichi Suzuki, Issei Komuro

**Affiliations:** 1 Department of Cardiovascular Medicine, Graduate School of Medicine, The University of Tokyo, 7-3-1 Hongo, Bunkyo-ku, Tokyo, 113-8655 Japan; 2 Department of Advanced Clinical Science and Therapeutics, Graduate School of Medicine, The University of Tokyo, Bunkyo-ku, Tokyo, 113-8655 Japan; 3 AMED-CREST, Japan Agency for Medical Research and Development, Chiyoda-ku, Tokyo, 100-0004 Japan

**Keywords:** AT_1_ receptor, ARB, Cardiovascular disease, Complement C1q, Skeletal muscle regeneration

## Abstract

The renin-angiotensin system (RAS) regulates diverse cellular responses and is crucial for normal organ development and function. On the other hand, RAS exerts deleterious effects promoting cardiovascular and multiple organ damage and contributes to promoting various aging-related diseases and aging-related decline in multiple organ functions. RAS blockade has been shown to prevent the progression of aging-related phenotypes and promote longevity. Wnt signaling pathway also plays a major role in the regulation of mammalian pathophysiology and is essential for organismal survival, and furthermore, it is substantially involved in the promotion of aging process. In this way, both RAS signaling and Wnt signaling have the functions of antagonistic pleiotropy during the process of growth and aging. Our recent study has demonstrated that an anti-aging effect of RAS blockade is associated with down-regulation of canonical Wnt signaling pathway, providing evidence for the hierarchical relationship between RAS signaling and Wnt signaling in promoting aging-related phenotypes. Here, we review how RAS signaling and Wnt signaling regulate the aging process and promote aging-related diseases.

## Background

The renin-angiotensin system (RAS) plays pleiotropic roles in regulating mammalian pathophysiology. Angiotensin II (Ang II) is a key molecule of RAS and is produced as a result of sequential cleavage of angiotensinogen by renin and angiotensin-converting enzyme (ACE). Ang II exerts diverse pathophysiological effects on binding to Ang II type 1 (AT_1_) receptor [1]. AT_1_ receptor is a well-known member of the G protein-coupled receptor family, which shares the structure characterized by seven transmembrane-spanning α-helices. Mice have two AT_1_ receptor isoforms (AT_1a_ and AT_1b_) which are encoded by separate genes (*Agtr1a* and *Agtr1b*), whereas humans have a single AT_1_ receptor isoform encoded by *AGTR1* gene. Mouse *Agtr1a* gene is a homolog to human *AGTR1* gene, and AT_1a_ receptor is the major AT_1_ receptor isoform in mice. AT_1_ receptor is activated by binding of Ang II or by mechanical stretch in the absence of Ang II [2, 3].

A decrease in extracellular volume caused by fluid loss or low salt intake stimulates secretion of renin, which leads to production of Ang II and thereby induces systemic vasoconstriction, salt and water retention, and sympathetic nervous activation. These responses restore blood pressure and electrolyte and water balance. In addition to regulation of hemodynamic homeostasis, RAS is essential for normal organ development. Mice deficient in angiotensinogen [4] or in both AT_1a_ and AT_1b_ receptor isoforms [5, 6] showed abnormal phenotypes in the kidney. The administration of ACE inhibitors or AT_1_ receptor blockers (ARBs) is contraindicated during pregnancy due to an increased risk of fetal disorders [7]. Thus, RAS is crucial for both embryogenesis and maintaining homeostasis and apparently beneficial for survival.

On the other hand, RAS has detrimental effects on cardiovascular tissues. AT_1_ receptor activation evokes diverse G protein-dependent and G protein-independent signaling pathways, leading to cell proliferation, hypertrophic responses, apoptosis, generation of reactive oxygen species (ROS), and tissue inflammation [8]. RAS has been shown to promote the pathophysiological processes of various aging-related disorders, including not only cardiovascular diseases and heart failure but also diabetes, chronic kidney disease, dementia, osteoporosis, and cancer [9]. Recent studies have demonstrated that inhibition of RAS prolongs the physiological aging process and promotes longevity in rodents [10], suggesting the involvement of RAS in the aging process per se.

In addition to AT_1_ receptor, Ang II type 2 (AT_2_) receptor is also a functional receptor with high affinity for Ang II [1]. AT_2_ receptor activation has vasodilatory, anti-proliferative, and anti-inflammatory effects, which counteract the effects of AT_1_ receptor signaling [1]. Thus, AT_2_ receptor signaling may provide cardiovascular protection and possibly prevent the progression of aging-related diseases. Ang II is cleaved by ACE2 to form another peptide Ang (1–7). This ACE2-Ang (1–7) axis, acting via another G protein-coupled receptor Mas, is also involved in vasodilatory, anti-fibrotic, and anti-inflammatory properties [11]. While the ACE-Ang II-AT_1_ receptor axis has been extensively studied, research on the role of ACE2-Ang (1–7)-Mas receptor axis in the aging process has been limited.

Wnt signaling pathway also regulates diverse cellular responses during embryogenesis and is required for normal development and function of organs [12]. On the other hand, canonical Wnt/β-catenin signaling is also involved in the aging process and promotes aging-related phenotypes [13, 14]. Accordingly, both RAS and Wnt signaling pathway have antagonistic and pleiotropic effects in the physiological process of growth and aging because they are essential and beneficial early in life but deleterious later in life [9].

We have recently reported that RAS blockade prevented the aging-related functional decline in skeletal muscle and that this anti-aging effect of RAS blockade was associated with down-regulation of Wnt/β-catenin signaling pathway [15]. These findings suggest the relationship between RAS signaling and Wnt/β-catenin signaling in promoting aging-related phenotypes. This review focuses on how RAS and Wnt signaling pathway regulate the aging process and how they play roles as possible targets for preventing and treating aging-related diseases.

## RAS in aging-related cardiovascular diseases and heart failure

Aging is usually defined as a progressive loss of multiple organ functions with advancing age. It is regulated by a wide variety of factors including genetic backgrounds and environmental stresses. Aging increases the risks of various cardiovascular diseases, such as hypertension, atherosclerotic vascular disease, cardiac remodeling, and congestive heart failure. RAS is widely recognized as a key factor contributing to the pathogenesis throughout the “cardiovascular continuum” [16].

RAS plays a fundamental role in the pathogenesis of hypertension even at an early stage. Sustained and excessive activation of RAS signaling induces continuous vasoconstriction and promotes vascular hypertrophy and endothelial dysfunction by direct as well as indirect hemodynamic effects, thereby contributing to the accelerated rise of blood pressure [17]. Indeed, the administration of an ARB reduced the development of hypertension in prehypertensive patients [18]. RAS blockade leads to a better outcome on survival in high-risk animals with hypertension. The treatment with an ACE inhibitor or ARB doubled the life span of stroke-prone spontaneously hypertensive rats to 30 months, which was comparable to that of normotensive rats [19, 20]. This life extension effect was associated with preservation of cardiac function as well as endothelial function by the treatment with an ACE inhibitor or ARB [19, 20].

RAS contributes to the promotion of atherosclerotic process. Ang II stimulates activity of nicotinamide adenine dinucleotide/nicotinamide adenine dinucleotide phosphate (NAD(P)H) oxidase which increased ROS formation [21]. Ang II also stimulates the release of proinflammatory cytokines and promotes the recruitment of macrophages and T cells through the generation of adhesion molecules and chemokines [22]. This increased oxidative stress and proinflammatory state leads to the development of endothelial dysfunction and vascular remodeling [23]. Furthermore, RAS promotes vasoconstriction, alters the composition of extracellular matrix, and enhances migration and proliferation of vascular smooth muscle cells [24]. Thus, RAS induces diverse cellular responses within the vascular wall, leading to the progression of atherosclerotic cascade.

Persistent and excessive activation of RAS plays a substantial role in pathological cardiac hypertrophy and cardiac remodeling. AT_1_ receptor activation induces hypertrophic responses in cardiomyocytes and extracellular matrix protein synthesis in cardiac fibroblasts [25]. Ang II infusion induced cardiac hypertrophy independently of blood pressure elevation in rats [26], and cardiac-specific overexpression of AT_1_ receptor induced cardiac hypertrophy, interstitial fibrosis, and contractile dysfunction in mice [27, 28]. RAS blockade is highly effective in preventing the progression of cardiac remodeling and heart failure. AT_1a_ receptor-deficient mice showed less severe cardiac dysfunction induced by myocardial infarction [29], administration of cardiotoxic agent doxorubicin [30], or genetic disruption of muscle LIM protein (MLP) [31]. A meta-analysis demonstrated that ARBs were the most effective among antihypertensive drugs for reducing left ventricular mass in patients with essential hypertension [32]. In addition, clinical trials have demonstrated that ACE inhibitors or ARBs reduce death and hospitalization in the broad spectrum of patients with heart failure [33].

## RAS in other aging-related diseases

It has been demonstrated that RAS is involved in the pathophysiological processes of other aging-related disorders, such as diabetes, chronic kidney disease, dementia, osteoporosis, and cancer [9].

RAS contributes to the pathogenesis of insulin resistance, a notable feature of metabolic syndrome and type 2 diabetes mellitus [34]. Ang II-mediated activation of AT_1_ receptor modulates the effects of insulin signaling [35]. For example, AT_1_ receptor activation synergistically promotes the proliferative effects of insulin but inhibits its metabolic actions. Also, the direct effect of Ang II on islet contributes to impaired β cell function. Acute infusion of Ang II inhibited the early phase of insulin secretion in rats [36], and RAS blockade improved islet morphology and prevented islet fibrosis in diabetic rats [37]. Recent clinical trials have demonstrated that RAS blockade improves insulin sensitivity and reduces the incident risk of diabetes in high-risk patients [38].

RAS signaling plays a major role in promoting chronic kidney disease. Ang II induces vasoconstriction of the post-glomerular arterioles and increases the glomerular hydrostatic pressure and the ultrafiltration of plasma proteins, thereby contributing to the onset and progression of chronic renal damage [39]. In addition to hemodynamic effects, Ang II exerts non-hemodynamic effects promoting renal tissue injury, through increased generation of ROS, up-regulation of cytokines and adhesion molecules, activation and recruitment of macrophages, and increased synthesis of extracellular matrix proteins [40]. RAS inhibitors provide renal protection and reduce proteinuria and decline of glomerular filtration rate in patients with chronic kidney disease [41, 42].

Recent studies unraveled the possible involvement of RAS in neuropathology of Alzheimer’s disease or bone metabolism. ARB treatment decreased the accumulation of β-amyloid proteins in the brain and attenuated the development of cognitive impairment in a mouse model of Alzheimer’s disease [43]. In ovariectomized rats with hypertension, ARB treatment attenuated osteoporosis and suppressed an increase in osteoclast activity [44]. Clinical studies have suggested that antihypertensive treatment with ACE inhibitors was associated with high bone mineral density and reduced the risk of bone fractures [45].

Furthermore, RAS is associated with cancer-related signaling pathways. AT_1_ receptor is expressed in several human cancer cell lines including pancreatic and prostate cancer cell lines [46, 47] and in a subpopulation of *ER*-positive, *ERBB2*-negative breast cancer cases [48], and ARB treatment suppressed AT_1_ receptor-positive cancer cell proliferation and tumor growth. The growth of tumor cells engrafted in AT_1a_ receptor-deficient mice was reduced, accompanied by reduction in tumor-related angiogenesis [49]. Ang II induced the proliferation of myeloid progenitor cells in the spleen in a mouse model of lung adenocarcinoma, thereby supplying tumor-associated macrophages to promote tumor development [50]. These findings indicate that RAS signaling plays an important role in tumor growth and progression through inducing tumor cell proliferation, tumor-associated angiogenesis, and tumor-associated macrophage expansion.

## RAS in physiological aging process

RAS blockade has been shown to suppress the deleterious effects during the physiological aging process in rodents. The administration of an ACE inhibitor or ARB to CF1 mice or Wistar rats led to a prolongation of life span, which was associated with a decrease in cardiac and renal fibrosis [51, 52]. CF1 mice treated with enalapril, but not with propranolol, nifedipine, or hydrochlorothiazide, revealed protection from organ damage associated with aging and prolonged life span, even though these drugs induced similar hypotensive effects [53]. Therefore, RAS inhibitor regulates life span independently of blood pressure-lowering effect. AT_1a_ receptor-deficient mice also exhibited a prolongation of life span, which was accompanied by less cardiac hypertrophy and fibrosis [10]. The accumulation of oxidative stress caused by ROS substantially contributes to the aging process [54]. The treatment with an ACE inhibitor was associated with a decrease in apoptosis and the suppression of aging-related decrease in mitochondrial number and mitochondrial superoxide dismutase in murine cardiomyocytes [51, 55]. AT_1a_ receptor-deficient mice showed less oxidative damage in the heart and kidney and prevention of aging-related loss of mitochondria in the kidney [10]. In addition, genetic disruption of AT_1a_ receptor induced an increase in expression levels of *Nampt* and *Sirt3* in the kidneys of aging mice [10]. In a nutrient-deprived environment, increased expression of *Nampt* leads to the accumulation of its biosynthetic product nicotinamide adenine dinucleotide (NAD^+^) in mitochondria, which in turn activates mitochondrial sirtuin 3 (SIRT3) [56]. SIRT3 is a NAD^+^-dependent deacetylase that protects against stress-mediated cell death. These findings demonstrate that inhibition of RAS promotes longevity, possibly through attenuation of oxidative stress and up-regulation of prosurvival genes.

We have recently reported that AT_1a_ receptor-deficient mice showed less severe aging-related phenotypes in other tissues as well, such as functional decline in skeletal muscle [15]. RAS blockade can prevent aging-related sarcopenia. It has been demonstrated that elderly persons with hypertension taking ACE inhibitors had lower decline in muscle strength and larger muscle mass of the lower extremities than users of other antihypertensive drugs [57, 58]. In aged mice, ARB treatment protected against disuse atrophy of skeletal muscle [59]. One of the hallmarks of aging is a decline in the regenerative capacity of skeletal muscle following injury. ARB treatment led to histological improvement in skeletal muscle regeneration after laceration in mice [60]. ARB inhibited transforming growth factor-β (TGF-β) signaling and thereby attenuated TGF-β-mediated impairment of muscle regeneration in mice with myopathy [61]. In addition, Ang II may have direct anti-proliferative effects on satellite cells, which are crucial for skeletal muscle growth and regeneration, via AT_1_ receptor [62]. We have shown that treatment with an ARB restored skeletal muscle function assessed by treadmill test after cryoinjury in mice [15]. ARB-treated mice showed an increase in satellite cell population, enhanced regeneration of myofibers, and decreased fibrosis in cryoinjured skeletal muscle. Taken together, RAS can be targeted to protect against deleterious effects associated with aging process and promote longevity.

## Relationship between RAS and Wnt signaling in aging

Wnt proteins initiate signaling cascade on binding to Frizzled receptor and low-density lipoprotein receptor-related protein (LRP) 5/6 coreceptor, which leads to stabilization of cytosolic β-catenin [12, 63]. Then, translocating to nucleus, β-catenin activates target gene transcription. It has been demonstrated that canonical Wnt/β-catenin signaling is also involved in the aging process. Wnt/β-catenin signaling activity was enhanced in multiple tissues of a klotho-deficient mouse model of accelerated aging [64]. Wnt treatment attenuated skeletal muscle regeneration in young mice, and inhibition of canonical Wnt signaling restored the impairment of muscle regeneration in aged mice [13]. Moreover, Wnt/β-catenin signaling was augmented in skeletal muscle satellite cells exposed to the serum of aged mice, indicating that components of aged serum contributed to the aging-related activation of Wnt signaling [13]. Complement C1q has recently been identified as an activator of Wnt/β-catenin signaling independently of Wnt [14]. C1q activates canonical Wnt/β-catenin signaling through binding to Frizzled receptor and inducing C1s-dependent cleavage of LRP6 coreceptor. Macrophages are major cells that secrete C1q [65]. Aging mice have increased serum and tissue levels of C1q and enhanced Wnt signaling activity [14]. C1q treatment stimulated fibroblast proliferation and collagen synthesis but suppressed satellite cell proliferation in skeletal muscle, and promoted aging-related impairment of skeletal muscle regeneration through activation of Wnt/β-catenin signaling [14]. C1q was secreted from macrophages recruited to the aorta in Ang II-infused mice, and C1q-mediated activation of Wnt/β-catenin signaling induced proliferation of vascular smooth muscle cells and promoted arterial remodeling [66].

**Fig. 1:**
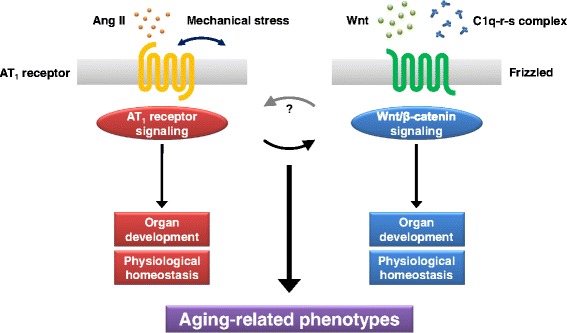
RAS signaling and Wnt signaling in aging process. AT_1_ receptor is activated upon stimulation by binding of Ang II or mechanical stress, and Wnt/β-catenin signaling cascade is initiated by binding of Wnt proteins or C1q-r-s complex to Frizzled receptor. Both AT_1_ receptor signaling and Wnt/β-catenin signaling are essential for normal organ development and crucial for regulation of physiological homeostasis. On the other hand, AT_1_ receptor signaling and Wnt/β-catenin signaling are involved in the aging process, and they are hierarchically related in promoting aging-related phenotypes. AT_1_ receptor blockade protects against aging-related deleterious effects through down-regulation of aging-promoting C1q-Wnt/β-catenin signaling pathway

We have demonstrated that serum C1q concentration was increased, and C1q expression and Wnt/β-catenin signaling activity in skeletal muscle were augmented after cryoinjury in mice, but ARB treatment inhibited both the increase in serum C1q level and the activation of C1q-Wnt/β-catenin signaling in injured muscle [15]. C1q expression in macrophages was reduced by administration of ARB both in culture and in injured muscle. Moreover, these beneficial effects of ARB on skeletal muscle repair after injury were reversed by topical administration of C1q [15]. These findings suggest that RAS blockade prevents aging-related phenotypes through down-regulation of aging-promoting C1q-Wnt/β-catenin signaling pathway and that RAS signaling and Wnt/β-catenin signaling are hierarchically related in promoting the aging process (Fig. 1). We propose that RAS blockade reduces systemic and local levels of C1q through inhibiting C1q synthesis in infiltrated macrophages and thereby enhances proliferation and differentiation of satellite cells, leading to promotion of skeletal muscle repair. Besides selectively inhibiting Ang II-induced activation of AT_1_ receptor, ARB may enhance Ang II-induced activation of AT_2_ receptor [67]. Further studies will be required to elucidate whether AT_2_ receptor activation contributes to down-regulation of C1q-Wnt/β-catenin signaling and protection against aging.

## Conclusions

RAS is profoundly involved in the progression of various aging-related diseases and the promotion of the aging process. RAS blockade has been shown to protect against aging-related deleterious effects and promote longevity. We have recently shown that this anti-aging effect of RAS blockade was mediated by down-regulation of aging-promoting C1q-Wnt/β-catenin signaling pathway, suggesting the hierarchical relationship between RAS signaling and Wnt signaling in promoting aging-related phenotypes. It is a matter of interest whether blocking RAS signaling or C1q-Wnt/β-catenin signaling could prevent geriatric frailty and prolong life span in humans. It remains to be elucidated how RAS signaling induces C1q expression in macrophages during the aging process. Further investigations of the relationship between RAS signaling and C1q-Wnt/β-catenin signaling will provide insights into the mechanisms responsible for aging-related functional decline of multiple organs and open up a path toward the development of novel therapeutics against aging-related diseases.

## Abbreviations

ACE, angiotensin-converting enzyme; Ang, angiotensin; ARB, angiotensin II type 1 receptor blocker; AT_1_, angiotensin II type 1; LRP, low-density lipoprotein receptor-related protein; MLP, muscle LIM protein; NAD(P)H, nicotinamide adenine dinucleotide/nicotinamide adenine dinucleotide phosphate; NAD^+^, nicotinamide adenine dinucleotide; RAS, renin-angiotensin system; ROS, reactive oxygen species; SIRT, sirtuin; TGF-β, transforming growth factor-β.
